# From observation to protection a bibliometric analysis of aerosol-glacier interactions

**DOI:** 10.1016/j.isci.2026.114672

**Published:** 2026-01-30

**Authors:** Hongfei Meng, Feiteng Wang, Shuangshuang Liu, Xiang Jin, Mengwei Xu, Jianxin Mu

**Affiliations:** 1 Xinjiang Tianshan Glacier National Field Observation and Research Station, Northwest Institute of Eco-Environment and Resources, Chinese Academy of Sciences, Lanzhou 730000, China; 2 University of Chinese Academy of Sciences, Beijing 100049, China

**Keywords:** Earth sciences, Glacial processes, Remote sensing

## Abstract

Remote sensing has become a central approach for investigating interactions between atmospheric aerosols and glacier change under climate warming. Here, we apply bibliometric and science-mapping methods to systematically analyze global research on remote sensing of aerosol-glacier interactions from 1995 to 2024. Based on 523 publications, we identify rapid growth since 2013 and reveal a tripolar collaboration structure linking North America, Europe, and Asia. Thematic analyses show a clear evolution from early observational and retrieval studies toward process-oriented research emphasizing black carbon deposition, snow-ice energy balance, and regional glacier mass balance. Journal dual-map overlays and cluster dependency analysis further demonstrate that the field is supported by Earth sciences, physics-chemistry, and computational systems, with aerosol type and scientific assessment serving as key foundational clusters. Overall, this study outlines the knowledge structure and evolution of aerosol-glacier research, supporting interdisciplinary monitoring and protection strategies in a changing climate.

## Highlights

• Bibliometric review of remote sensing studies on aerosol-glacier interactions (1995–2024)
• Rapid growth since 2013 with a tripolar “North America–Europe–Asia” collaboration structure
• Research hotspots shifted to black carbon, glacier mass balance, and climate impacts
• Provides a knowledge framework supporting cryosphere monitoring and preservation strategies

## Graphical abstract

**Figure:**
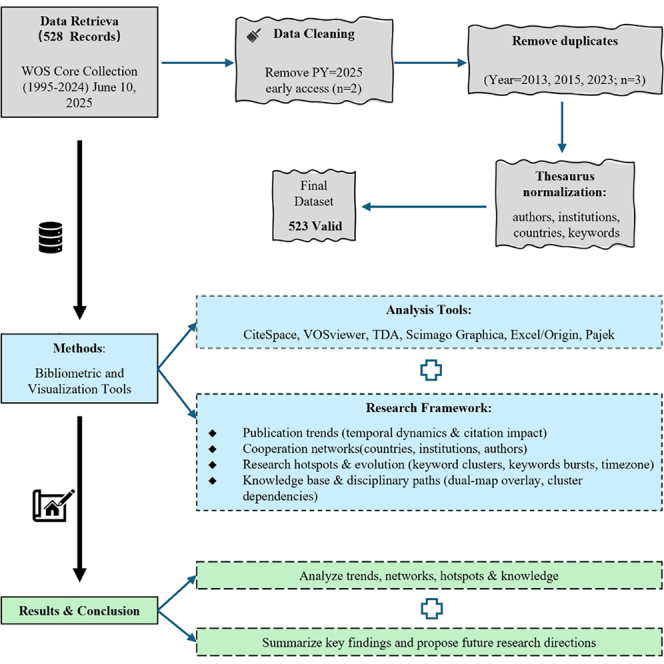

## Introduction

Glaciers are among the most sensitive indicators of global climate change. Their melting not only directly contributes to sea-level rise but also exerts profound impacts on water security in arid regions.^1^ Under the context of global warming, atmospheric aerosols have emerged as a key external driver influencing glacier change.^2^ Through radiative effects and deposition processes, aerosols alter the surface albedo and energy balance of snow and ice, accelerating melt processes.^3^ Among them, light-absorbing aerosols such as black carbon and mineral dust have attracted particular attention for their role in enhancing glacier ablation and modifying regional and global climate systems.^4^^,^^5^ Consequently, exploring the interactions between aerosols and glacier dynamics has become a frontier topic of climate change and water resources research.^6^^,^^7^

Compared with conventional *in situ* observations, remote sensing technologies offer advantages of extensive spatial coverage, long-term time series, and multi-scale monitoring, making them indispensable tools in investigating aerosol-glacier interactions.^8^^,^^9^ Satellite sensors (e.g., MODIS, MERIS, CALIPSO, VIIRS) and ground-based remote sensing instruments (e.g., lidar and radiometers) have provided critical datasets for studying aerosol optical properties, transport mechanisms, and deposition processes, as well as glacier mass balance, snow cover, and surface energy fluxes.^10^^,^^11^^,^^12^ In recent years, the integration of multi-source remote sensing data and the advancement of climate models have fostered a comprehensive framework that combines observation, simulation, and application.^13^^,^^14^ However, most existing studies remain focused on specific cases or regions, while systematic analyses from a knowledge-structure and evolutionary perspective are still limited. In this interdisciplinary domain, research outputs remain fragmented across remote sensing, atmospheric, and cryospheric sciences, making it difficult to capture how collaborations and themes have evolved over time. Bibliometric and science-mapping approaches address this gap by providing quantitative and reproducible insights into co-authorship structures, thematic clustering, and temporal evolution. Recent bibliometric studies on glacier-aerosol-hydrology research have also examined these interactions from different perspectives, further demonstrating the practical value and methodological significance of bibliometric analysis in understanding the development of glacier-related environmental studies.^15^^,^^16^ Unlike narrative reviews, these methods reveal the intellectual organization and development trajectory of the field, offering an evidence-based foundation for advancing observation, modeling, and protection strategies.

To address these limitations and reveal the structural evolution of this interdisciplinary domain, the present study employs bibliometric and visualization approaches to systematically examine the application of remote sensing technologies in aerosol-glacier research. By analyzing relevant publications between 1995 and 2024, we reveal the research status, cooperative networks, knowledge bases, and frontier hotspots of the field, and further identify the thematic evolution paths and dependency relationships across clusters. Unlike traditional narrative reviews, this work emphasizes the quantitative exploration of literature structures, providing a holistic perspective on the development trajectory of aerosol–glacier studies. Furthermore, recent advances in bibliometric analysis have shown its potential to connect scientific evidence with sustainability and policy frameworks, providing a methodological foundation for translating observation-based research into protection-oriented decision-making.^17^ By linking the observation-based research framework with the needs of glacier protection and sustainable cryosphere management, this study also aims to bridge scientific monitoring and policy-oriented decision-making, aligning with the concept of “From Observation to Protection.” The findings not only clarify the knowledge framework of the field but also offer guidance for advancing future research in remote sensing monitoring, mechanism modeling, and regional applications.

## Results and discussion

### Publication trends and impact

#### Research trends

**Figure 1:**
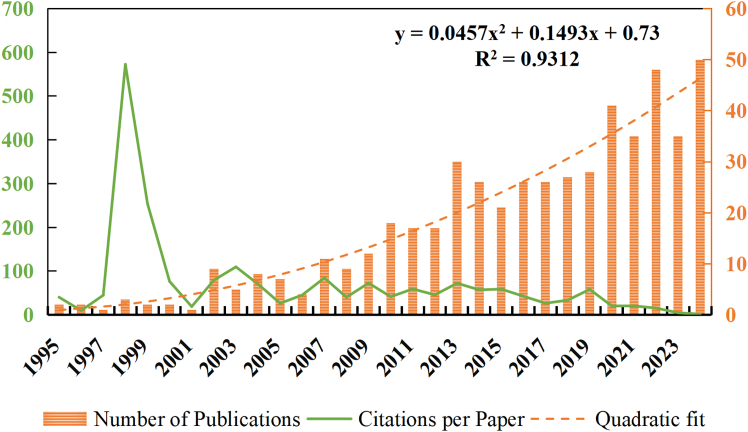
Research trends in remote sensing studies on aerosol-glacier interactions (1995–2024) This figure shows the annual number of publications and the average citations per article related to remote sensing studies on aerosol-glacier interactions from 1995 to 2024. The solid line represents the annual publication output, while bars indicate the average citations per article. A quadratic fitting curve is used to illustrate the long-term growth trend of publications, with the coefficient of determination (R^2^) indicating the goodness of fit. Overall, this figure demonstrates the sustained and accelerating growth of aerosol-glacier remote sensing research over the past three decades.

As shown in Figure 1, the development of remote sensing studies on aerosol-glacier interactions from 1995 to 2024 can be divided into three main stages. During 1995–2006, the field was in its initial exploratory stage, with fewer than 10 articles per year and a relatively high average citation rate (about 112.5 citations per article). In this period, a few pioneering works published in 1998 and 1999 each received over 250 citations, laying the theoretical and methodological foundation for subsequent studies. From 2007 to 2019, the research entered a steady growth stage, with an average of 20.6 articles published annually and a moderate average citation of 53.4 per article. This stage reflects the gradual expansion of research activity and the establishment of a more stable publication pattern as remote sensing and atmospheric monitoring technologies improved. Since 2020, the annual publication output has further increased to an average of 41.8 articles, reaching 35–50 per year, while the average citation per article declined to about 12.7 due to the rapid accumulation of new outputs and shorter citation windows. The quadratic fitting (R^2^ = 0.9312) demonstrates a significant long-term upward trend, indicating that studies on aerosol-glacier interactions using remote sensing have evolved from scattered exploratory efforts into a systematic and steadily growing research field.

#### Highly cited publications (top 10)

The ten highly cited articles span the period from 1998 to 2019, reflecting the complete process of development in this field—from sensor design to climate model advancement (Table 1). Early studies focused on sensor performance and algorithm development, such as the proposal of the MODIS cloud detection algorithm (1998),^16^ the prelaunch characterization of MODIS (1998),^17^ and the review of the MERIS instrument and mission (1999),^22^ which laid the data foundation for subsequent remote sensing applications of aerosols and the cryosphere. Entering the 2000s, research gradually expanded to extreme events and regional environmental applications, such as the “Arctic smoke” study that first revealed the severe impacts of agricultural burning on Arctic air pollution and snow albedo (2007),^21^ and precipitation observations and future scenarios in the Hindu-Kush-Karakoram-Himalaya region (2013).^19^ Since 2010, research has increasingly shifted toward global observations and model simulations, including the on-orbit performance evaluation of VIIRS (2014),^28^ the EarthCARE satellite mission (2015),^29^ the ESA Climate Change Initiative (2013),^18^ and the development of the WACCM6 model (2019).^20^ In addition, a 2016 review provided a systematic synthesis of the uncertainties in aerosol-cloud interactions,^30^ which has become a key reference in the field.

**Table 1:** Top 10 highly cited publications on remote sensing of aerosols and glaciers (1995–2024)

Title	Publication year	Total citations
Discriminating clear sky from clouds with modis^18^	1998	981
Prelaunch characteristics of the moderate resolution imaging spectroradiometer (modis) on eos-am1^19^	1998	712
Improving our fundamental understanding of the role of aerosol-cloud interactions in the climate system^20^	2016	527
Early on-orbit performance of the visible infrared imaging radiometer suite onboard the Suomi National Polar-Orbiting Partnership (s-npp) satellite^21^	2014	441
The earthcare satellite the next step forward in global measurements of clouds, aerosols, precipitation, and radiation^22^	2015	427
The esa climate change initiative satellite data records for essential climate variables^23^	2013	374
Precipitation in the Hindu-Kush Karakoram Himalaya: observations and future scenarios^24^	2013	366
The whole atmosphere community climate model version 6 (waccm6)^25^	2019	365
Arctic smoke -: record high air pollution levels in the European Arctic due to agricultural fires in eastern Europe in spring 2006^26^	2007	322
The esa medium resolution imaging spectrometer meris -: A Review of the Instrument and its mission^27^	1999	313

From the perspective of research content, these highly cited publications can be grouped into three major categories. The first is instrument- and data product-oriented studies, such as those on MODIS,^16^^,^^17^ MERIS,^22^ VIIRS,^28^ and EarthCARE,^29^ which provide long-term, continuous, and multi-variable observational foundations for aerosol and glacier change studies. The second is project- and program-related outputs, such as ESA CCI,^18^ which established standardized datasets for climate research by reprocessing and integrating satellite data. The third is model- and mechanism-focused studies, including the aerosol-cloud interaction review,^30^ the development of WACCM6,^20^ and regional case studies,^19^^,^^21^ which promoted close integration between observations, datasets, and climate simulations. Collectively, these works established a complete chain linking “data products—observation missions—model development—application validation.”

Overall, the Top 10 highly cited articles highlight three key characteristics of the field. First, technology-driven progress: early highly cited studies concentrated on sensor development and data products, providing a solid foundation for subsequent research. Second, multi-scale integration: the literature encompasses both global-scale observation missions and modeling efforts,^18^^,^^20^^,^^29^^,^^30^ as well as representative regional and event-based studies.^19^^,^^21^ Third, knowledge system construction: through the stepwise linkage of sensors, datasets, models, and applications, these studies collectively established the international academic framework for research on aerosols and glacier change. Their high citation counts not only demonstrate the broad applicability of their methods and datasets but also underscore the community’s reliance on interdisciplinary and cross-scale integration.

### Cooperation networks

#### National and regional cooperation

**Figure 2:**
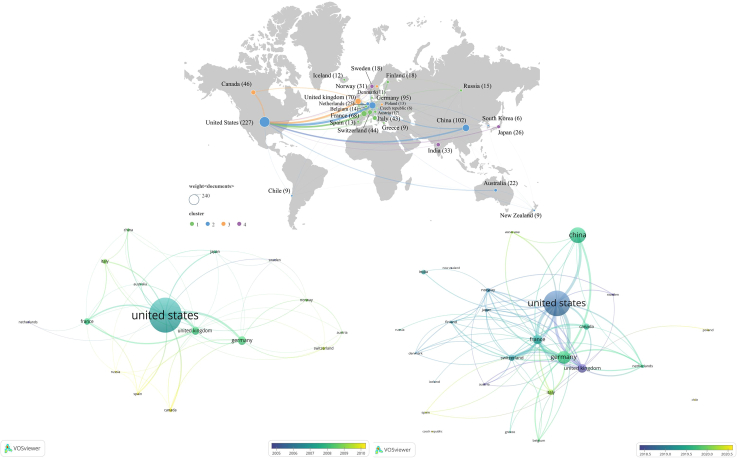
National and regional cooperation network in remote sensing studies on aerosol-glacier interactions (1995–2024) This map-based network illustrates international collaboration patterns based on co-authored publications. Nodes represent countries or regions, with node size proportional to publication output. Links indicate collaborative relationships, and link thickness reflects collaboration strength. Node colors denote different collaboration clusters identified through network analysis. Overall, this figure highlights a tripolar collaboration structure centered on North America, Europe, and Asia. (A and B) National collaboration networks during 1995–2012 (2a, left) and 2013–2024 (2b, right). These panels compare national collaboration patterns between the early stage (1995–2012) and the recent stage (2013–2024) of aerosol-glacier remote sensing research, using consistent visualization rules. Differences in network density and connectivity reflect the temporal evolution of international cooperation. Overall, this figure shows a clear expansion and densification of global collaboration after 2013.

Among the countries with ≥20 publications, the United States leads with 227 articles, serving as the global core force in this field. Canada, with 46 publications, also plays an important role, forming a strong North American research hub. European countries demonstrate overall strength, with Germany (95), the United Kingdom (70), France (68), Switzerland (44), Italy (43), the Netherlands (25), and Norway (31) forming a relatively cohesive research cluster. In Asia, China ranks second globally with 102 publications, while India (33) and Japan (26) also make important contributions. In Oceania, Australia stands out with 22 publications, positioning itself among the major research countries (Table 2). The cooperation network (Figure 2) reveals a “North America–Europe–Asia” tripolar structure, with the United States maintaining close collaborations with Canada, the United Kingdom, Germany, and France, while China shows frequent cooperation with the United States, Germany, the United Kingdom, India, and Japan. To further demonstrate the temporal evolution of this network, the dataset was divided into two stages—1995–2012 (Figure 2A) and 2013–2024 (Figure 2B)—based on publication trends. The first period corresponds to the early development phase, when annual publications were generally below 20, and collaborations were mostly concentrated within a US-Western Europe core. In contrast, during 2013–2024, publication activity increased markedly, and the collaboration network became denser and more globally connected, with Asian countries—especially China—showing stronger engagement and expanded linkages with established research hubs. This evolution indicates that the global research on aerosol-glacier interactions has gradually shifted toward broader and more balanced international collaboration. In terms of collaboration intensity, the United States (TLS = 261), Germany (219), France (187), the United Kingdom (173), and Switzerland (130) exhibit the highest total link strengths in the international cooperation network, highlighting their roles as the major global hubs connecting regional research clusters. In addition, the H-index values further reflect national research influence, with the United States (H = 57) and Germany (H = 35) ranking highest, followed by the United Kingdom (H = 31) and China (H = 31), indicating the overall dominance of North America and Europe in citation impact.

**Table 2:** Publications, citations, average citations, average publication year, and H-index of countries/regions with ≥20 documents (1995–2024)

Countries	Documents	Total citations	Average citations per article	Average publication year	H-index
United States	227	16398	72.24	2014.98	57
China	102	7836	76.82	2018.85	31
Germany	95	8778	92.4	2017.52	35
United Kingdom	70	9117	130.24	2015.74	31
France	68	3667	53.93	2016.99	29
Canada	46	2026	44.04	2017.85	21
Switzerland	44	6385	145.11	2017.59	24
Italy	43	1738	40.42	2017.49	21
India	33	4997	151.42	2017.61	16
Norway	31	6008	193.81	2016.97	19
Japan	26	6182	237.77	2016.38	18
Netherlands	25	1519	60.76	2016.60	16
Australia	22	1037	47.14	2017.36	14

In terms of citation performance, the United States holds a dominant position with 16,398 total citations, highlighting its academic leadership. Several European countries exhibit high levels of average citations, such as the United Kingdom (130.24), Switzerland (145.11), Norway (193.81), and Germany (92.4), underscoring Europe’s strength in research depth and quality. Among Asian countries, Japan demonstrates exceptional academic influence, with an average citation count of 237.77 despite its relatively small number of publications, while India also performs well with an average of 151.42. By contrast, China’s average citation count (76.82) is lower than that of most European and some Asian countries; however, its total citation count has already reached 7,836, indicating that its academic influence is rapidly accumulating. Europe and North America continue to lead in terms of depth and impact, while China exhibits the characteristics of “high growth potential and enhanced international collaboration.”

The average year of publication reveals the entry timing and research activity levels of different countries. The United States, with an average publication year of 2014.98, is one of the earliest countries to enter the field and has remained highly active. Most European countries entered relatively early, with the United Kingdom (2015.74), France (2016.99), Norway (2016.97), the Netherlands (2016.60), Germany (2017.52), and Switzerland (2017.59) entering a phase of rapid development around 2015. Canada’s average publication year is 2017.85, slightly later than Europe, but as the second most important participant in North America, its research activity continues to rise. Asian countries present a differentiated pattern: Japan (2016.38) entered relatively early, while China (2018.85) and India (2017.61) significantly increased their contributions in the past decade. In Oceania, Australia (2017.36) also reflects accelerated growth in recent years. The field demonstrates a temporal pattern of “early entry and deep accumulation in Europe and North America, earlier participation from countries such as Japan, and rapid late-stage growth from emerging countries such as China.”

#### Institutional cooperation

**Figure 3:**
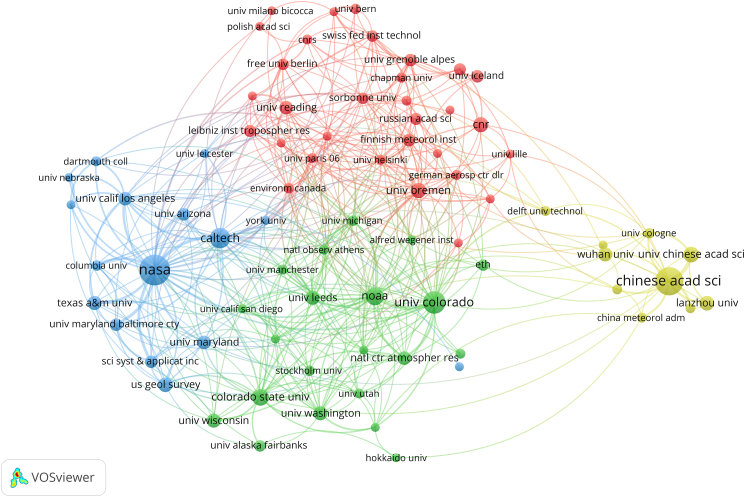
Institutional cooperation network in remote sensing studies on aerosol-glacier interactions (1995–2024) This network depicts collaboration among research institutions based on co-authorship. Nodes represent institutions, with node size proportional to publication output, while links indicate inter-institutional collaboration. Link thickness reflects collaboration intensity, and colors represent institutional clusters. Overall, this figure indicates that a limited number of core institutions act as major hubs in the global collaboration network.

Among institutions with ≥12 publications (Table 3), NASA (56) in the United States, together with Caltech (25), NOAA (19), and Colorado State University (17), form the core nodes of research collaboration. In the cooperation network, these institutions maintain close connections with multiple North American and European universities, highlighting the concentrated advantage of U.S. institutions in this field. In terms of collaboration intensity, NASA (TLS = 108), NOAA (66), the University of Colorado (59), Caltech (55), and the Chinese Academy of Sciences (49) exhibit the highest total link strengths in the institutional collaboration network, indicating their key roles as core nodes bridging major regional clusters. On the Chinese side, the Chinese Academy of Sciences (CAS) ranks second with 48 publications and forms a collaborative cluster with domestic institutions such as Wuhan University, Lanzhou University, and the China Meteorological Administration. Although Chinese institutions exhibit a tendency toward “independent clustering” in the collaboration network, they also maintain certain connections with core institutions in Europe and North America. European and North American research universities also show strong representation, including the University of Colorado (30), the University of Wisconsin (12), the University of Leeds (12), and the University of Washington (12), reflecting the broad scope of international collaboration (Figure 3). H-index analysis highlights that NASA (H = 33) and the Chinese Academy of Sciences (H = 24) possess the strongest institutional impact, followed by the University of Colorado (H = 20) and Caltech (H = 16), illustrating the sustained academic influence of leading U.S. and Chinese institutions in this field.

**Table 3:** Publications, citations, average citations, average publication year, and H-index of institutions with ≥12 documents (1995–2024)

Institutions	Documents	Total citations	Average citations per article	Average publication year	H-index
NASA	56	4104	73.29	2012.29	33
Chinese Acad Sci	48	1843	38.40	2018.29	24
Univ Colorado	30	1220	40.67	2015.10	20
Caltech	25	1690	67.60	2013.36	16
NOAA	19	1587	83.53	2017.11	18
Colorado State Univ	17	1991	117.12	2015.59	12
Univ Chinese Acad Sci	15	327	21.80	2020.47	10
Univ Bremen	14	575	41.07	2019.71	11
Cnr	14	469	33.50	2017.71	9
Us Geol Survey	13	758	58.31	2015.92	10
Lanzhou Univ	13	319	24.54	2020.23	10
Univ Wisconsin	12	2092	174.33	2011.33	11
Univ Leeds	12	1280	106.67	2016.33	10
Univ of Washington	12	1023	85.25	2017.58	9
Texas A&M University	12	490	40.83	2014.83	10
Wuhan Univ	12	245	20.42	2019.75	9

In terms of total citations, NASA ranks first with 4,104 citations. Caltech (1,690), NOAA (1,587), Colorado State University (1,991), the University of Wisconsin (2,092), and the University of Leeds (1,280) each exceed 1,000 citations, underscoring the leading academic influence of North American and European institutions. Average citations per publication also provide insights into academic impact: the University of Wisconsin (174.33), Colorado State University (117.12), the University of Leeds (106.67), and the University of Washington (85.25) all demonstrate high levels, whereas CAS (38.40), Wuhan University (20.42), Lanzhou University (24.54), and the University of Chinese Academy of Sciences (21.80) remain relatively lower. This indicates that while Chinese institutions are rapidly increasing their research output, their citation accumulation is still in a growth phase.

The average publication year further reveals the entry timing and research activity of each institution. Earlier entrants include the University of Wisconsin (2011.33), NASA (2012.29), and Caltech (2013.36), reflecting the long-term and sustained involvement of U.S. research institutions in the field. European institutions such as the University of Leeds (2016.33), the University of Washington (2017.58), and CNR (2017.71) became increasingly active after 2015. Chinese institutions generally entered later, with CAS (2018.29), Lanzhou University (2020.23), Wuhan University (2019.75), and the University of Chinese Academy of Sciences (2020.47) showing accelerated growth in recent years, indicating clear latecomer advantages and strong development potential.

#### Author cooperation

**Figure 4:**
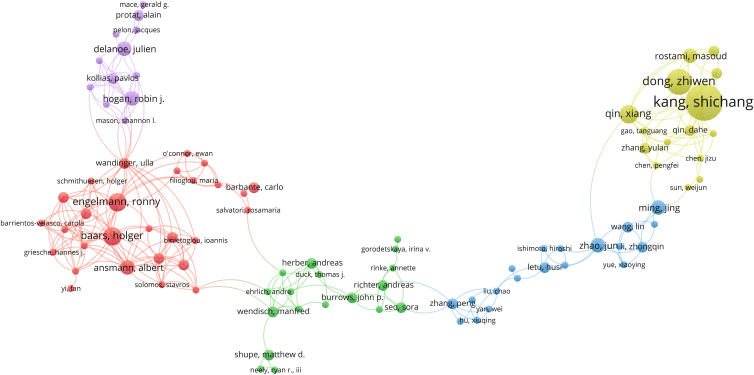
Author cooperation network in remote sensing studies on aerosol-glacier interactions (1995–2024) This figure shows co-authorship relationships among authors in the field. Nodes represent authors and links represent collaborative publications. Node size reflects publication output, and link thickness indicates collaboration strength. Colors identify author collaboration clusters. Overall, this figure reveals regionally clustered but internationally connected author collaboration patterns.

Among authors with ≥4 publications (Table 4), Chinese scholars have contributed a substantial number of outputs. “Kang Shichang ranks first with 10 publications and serves as a leading figure in aerosol-cryosphere research, focusing on the impacts of black carbon and mineral dust on glacier mass balance, albedo, and hydrological processes over the Tibetan Plateau and Central Asia. He and his team form the core of the Chinese collaborative cluster, working closely with Dong Zhiwen, Qin Xiang, and Zhao Jun. In the author network (Figure 4), this cluster shows strong internal links and active international connections, reflecting its central role in bridging regional research communities. In the cooperation network, this cluster shows strong internal connections, indicating a high degree of team collaboration. In terms of collaboration intensity, Kang Shichang (TLS = 34), Baars Holger (30), Engelmann Ronny (29), Dong Zhiwen (28), and Ansmann Albert (24) exhibit the highest total link strengths in the author collaboration network, reflecting their central roles in linking Chinese and European research communities. European scholars also form a distinct cooperation circle, including Baars Holger (5), Engelmann Ronny (5), Ansmann Albert (4), Delanoe Julien (4), and Hogan Robin J. (4), with relatively stable collaborative relationships. The author cooperation network presents a pattern of “regional clusters with inter-cluster connections,” in which Chinese and European scholar groups occupy central positions and are linked across regions through certain key individuals (Figure 4).

**Table 4:** Publications, citations, average citations, and average publication year of authors with ≥4 documents (1995–2024)

Authors	Documents	Total citations	Average citations per article	Average publication year
Kang, Shichang	10	395	39.5	2016.60
Dong, Zhiwen	7	240	34.29	2017.86
Baars, Holger	5	87	17.4	2021.00
Engelmann, Ronny	5	97	19.4	2021.00
Qin, Xiang	5	161	32.2	2017.20
Ansmann, Albert	4	195	48.75	2018.50
Delanoe, Julien	4	197	49.25	2016.25
Hogan, Robin J.	4	89	22.25	2020.25
Ming, Jing	4	79	19.75	2017.50
Rostami, Masoud	4	99	24.75	2019.75
Zhao, Jun	4	88	22	2019.75

In terms of citation performance, there are notable differences in academic impact among authors. Some scholars achieve both high total and average citation counts, such as Delanoe Julien (197 total citations, 49.25 per article) and Ansmann Albert (195, 48.75), whose work has received significant attention. Chinese scholars, while having larger publication outputs, generally exhibit moderate average citation levels, such as Kang Shichang (395, 39.5), Dong Zhiwen (240, 34.29), and Qin Xiang (161, 32.2). Authors with relatively lower average citations include Baars Holger (87, 17.4), Engelmann Ronny (97, 19.4), and Ming Jing (79, 19.75), which may be attributable to the relatively recent publication of their work, limited time for citation accumulation, or smaller collaboration networks.

The average year of publication reveals the temporal distribution of author activity. Some scholars entered the field earlier, such as Delanoe Julien (2016.25) and Kang Shichang (2016.60), indicating sustained contributions from an early stage. Around 2017, a group of scholars became active, including Qin Xiang (2017.20), Dong Zhiwen (2017.86), and Ming Jing (2017.50). After 2019, more recently active authors emerged, such as Zhao Jun and Rostami Masoud (both 2019.75), as well as European scholars such as Baars Holger and Engelmann Ronny (both 2021.00) and Hogan Robin J. (2020.25), reflecting the rapid growth of a new generation of researchers. Overall, the field demonstrates a developmental trajectory of “early contributors with sustained output—mid-stage scholars becoming active—emerging researchers rising rapidly.”

### Research hotspots and knowledge structure

#### Keyword clustering

##### Cluster ① | Observation methods and monitoring technologies

**Figure 5:**
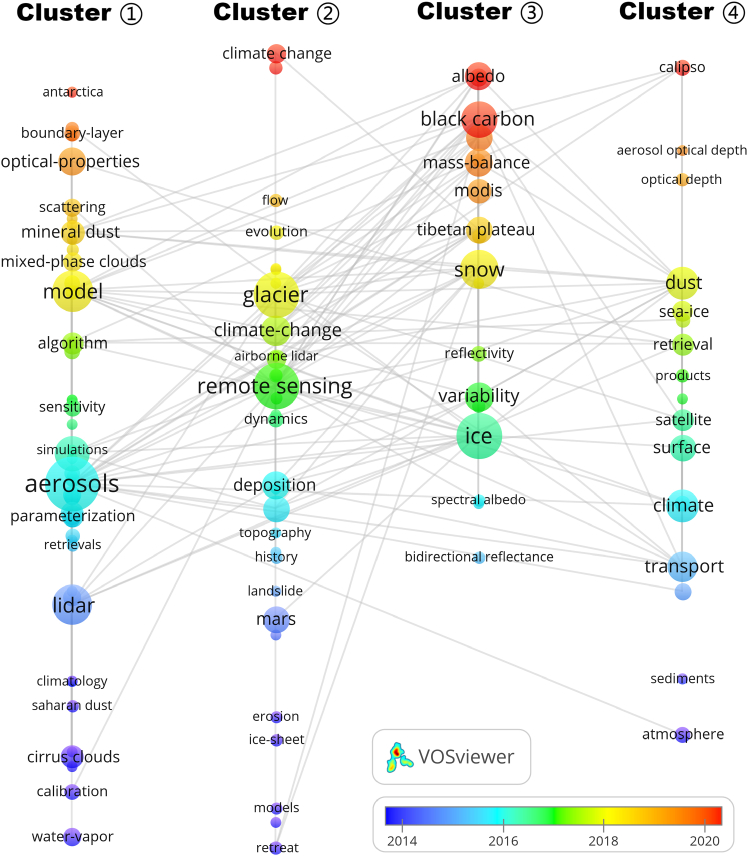
Keyword clustering of remote sensing studies on aerosol-glacier interactions (1995–2024). (color bar represents the average year of appearance of each keyword, from 2014 to 2020) This keyword co-occurrence network identifies major thematic clusters in the literature. Node size reflects keyword frequency, and links indicate co-occurrence relationships. Node colors represent the average year of appearance (AAY), illustrating the temporal evolution of research themes. AAY, average appearing year. Overall, this figure shows a progression from early observational methods toward process-oriented and energy-balance studies.

This cluster centers on remote-sensing/retrieval techniques and aerosol-cloud monitoring. High-frequency terms include lidar, aerosols, clouds, and models. Along the average-year gradient (Figure 5), early terms such as water vapor, calibration, and cirrus clouds reflect sensor calibration and basic atmospheric parameters; the middle stage highlights lidar, retrievals, and precipitation; more recent terms (e.g., algorithm, optical properties, boundary layer, and climate change) indicate growing links between retrieval methods, optical properties, and boundary-layer processes.

##### Cluster ② | Glacier processes and topography-deposition coupling

Representative terms include remote sensing, glacier, and deposition. Earlier keywords (e.g., retreat, ice sheet, erosion, and landslide) emphasize glacier change and geomorphic processes; the middle stage stresses topography, deposition, and remote sensing; recent terms (e.g., glacier, dynamics, flow, evolution) indicate a shift toward process-based analyses of glacier dynamics in typical mountain-polar environments.

##### Cluster ③ | Snow-ice energy balance and light-absorbing impurities

Key terms include ice, snow, variability, Tibetan Plateau, mass balance, black carbon, and albedo. Early work on bidirectional reflectance and spectral albedo points to radiative characterization; the middle stage focuses on ice/snow variability; recent terms such as Tibetan Plateau, MODIS, mass balance, and black carbon highlight the role of light-absorbing impurities in albedo reduction and mass-balance change.

##### Cluster ④ | Large-scale dust/AOD retrievals and polar linkages

Typical terms include dust, aerosol optical depth, optical depth, and CALIPSO. Early items (e.g., atmosphere, transport, and ice core) indicate background signals and transport; the middle stage emphasizes satellite products, retrieval, and surface/climate links; recent terms (dust, sea ice, AOD, and CALIPSO) show intensified use of multi-mission observations to connect long-range dust and cryospheric change.

In co-occurrence intensity, leading keywords include aerosols (TLS = 302), ice (223), snow (215), glacier (205), remote sensing (205), model (202), black carbon (196), lidar (186), clouds (161), and dust (155). The four clusters delineate a progression from early observation and retrieval development (Cluster ①), to glacier-topography-deposition coupling (Cluster ②), to snow-ice energetics influenced by light-absorbing impurities (Cluster ③), and finally to global dust/AOD retrievals with polar connections (Cluster ④).

In addition, the thematic evolution reflected by these clusters is broadly aligned with major international climate and Earth-observation initiatives. Cluster ① corresponds to efforts such as ESA’s Climate Change Initiative (CCI) and NASA Earthdata, which provide harmonized Essential Climate Variable (ECV) datasets for aerosols, clouds, and snow/ice. Clusters ② and ③ overlap with IPCC assessment themes on radiative forcing, cryosphere feedbacks, and surface-atmosphere interactions, where long-term open datasets are frequently used for model evaluation and trend detection. Cluster ④ echoes the integration of multi-mission satellite archives (e.g., MODIS, CALIPSO, Sentinel, and CryoSat) that support global data-sharing frameworks and facilitate large-scale analyses of dust transport, AOD patterns, and cryospheric change. Together, these connections illustrate that bibliometric hotspots have co-evolved with the increasing availability of standardized datasets, reproducible methodologies, and policy-relevant observational infrastructures in aerosol-cryosphere research.

#### Temporal evolution of hotspots

**Figure 6:**
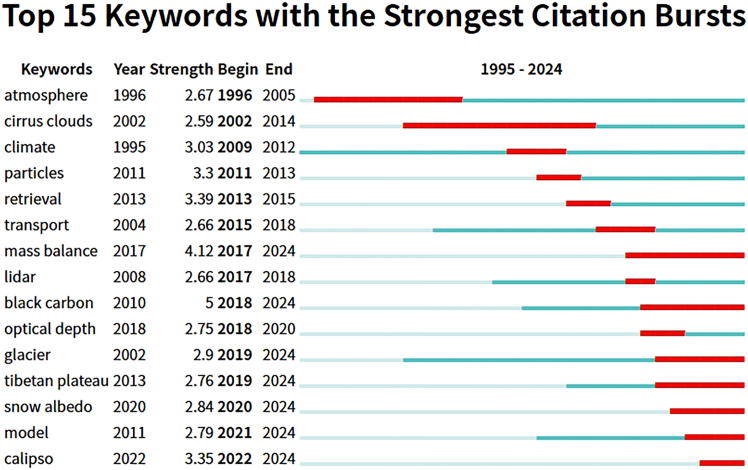
Top 15 keywords with the strongest citation bursts in remote sensing studies on aerosol-glacier interactions (1995–2024) This figure presents keywords that experienced rapid increases in citation frequency during specific time periods. Each bar represents the full study period, while highlighted segments indicate burst intervals. Overall, this figure identifies emerging research frontiers and shifting hotspots over time.

From the perspective of temporal evolution, the burstness analysis indicates a shift of research hotspots from fundamental concepts to regional and process-oriented investigations (Figure 6). Early burst keywords include atmosphere (1996–2005), cirrus clouds (2002–2014), and climate (2009–2012), reflecting that the initial stage of the field primarily focused on atmospheric characteristics, cloud microphysical properties, and climate system-related issues. In the past decade, burst keywords have increasingly shifted toward regional and process-driven topics such as mass balance (2017–2024), glacier (2019–2024), Tibetan Plateau (2019–2024), and snow albedo (2020–2024), highlighting growing attention to glacier change, energy balance, and environmental effects over the Tibetan Plateau. Meanwhile, technical terms including lidar (2017–2018), model (2021–2024), and CALIPSO (2022–2024) demonstrate the rapid integration of remote-sensing observations and model simulations in this field.

**Figure 7:**
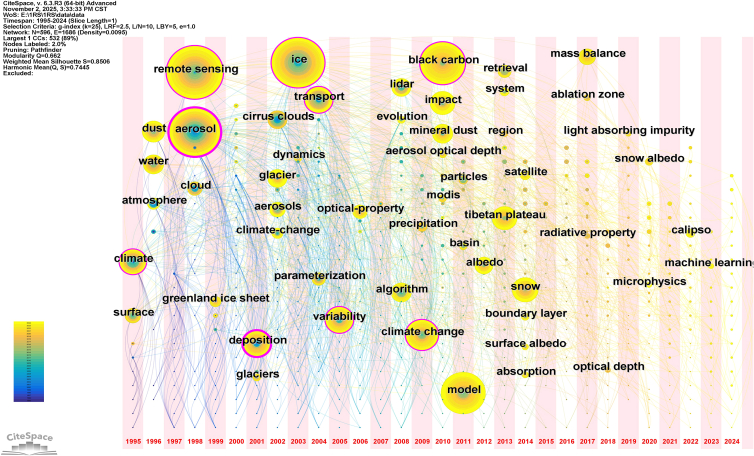
Keyword time zone view of remote sensing studies on aerosol-glacier interactions (1995–2024) This time zone visualization arranges keywords along a temporal axis to illustrate their emergence, persistence, and interconnections over time. Network modularity (Q) and mean silhouette value (S) indicate the quality and consistency of the identified clusters. Q, modularity; S, silhouette value. Overall, this figure illustrates the chronological shift from atmospheric observation toward regional and process-based glacier studies.

To better illustrate the evolution of research themes over time, the keyword timezone visualization (Figure 7) further reveals the chronological distribution and interlinkages of research clusters from 1995 to 2024. The network shows a high modularity (Q = 0.8506) and mean silhouette (S = 0.7445), indicating that the identified clusters are both clearly delineated and internally consistent. Early years are dominated by observation- and retrieval-related topics, followed by a middle phase characterized by intensified research on aerosol-cryosphere processes, and a recent stage emphasizing regional energy balance, black-carbon deposition, and Tibetan Plateau dynamics. This sequential pattern is consistent with the burstness findings and confirms a progressive evolution from atmospheric observation toward integrated studies of glacier-aerosol interactions and climatic impacts. Together, Figures 6 and 7 demonstrate a clear thematic transition from foundational atmospheric investigations to comprehensive, process-based, and regionally focused research frameworks.

#### Journal dual-map overlay

**Figure 8:**
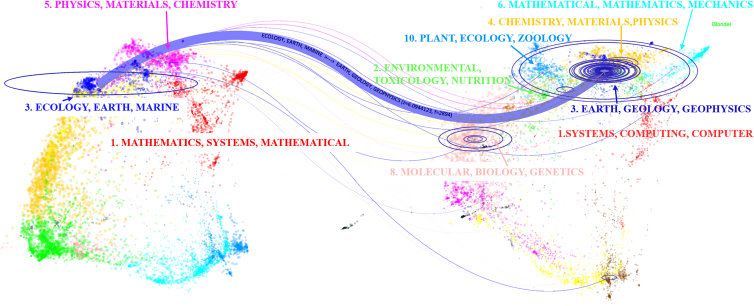
Journal dual-map overlay of remote sensing studies on aerosol-glacier interactions (1995–2024) The dual-map overlay shows citation trajectories between citing journals (left) and cited journals (right). Colored curves represent dominant knowledge flows across disciplinary domains, illustrating the interdisciplinary foundations of aerosol-glacier research. Overall, this figure demonstrates that the field is jointly supported by Earth sciences, physics-chemistry, and computational disciplines.

Figure 8 presents the journal dual-map overlay of aerosol-glacier remote sensing research, in which only the major journal subject clusters and the most prominent citation paths are annotated to enhance visual clarity. The left panel represents the disciplinary distribution of citing journals, while the right panel shows the subject clusters of cited journals, and the curved paths indicate the dominant citation trajectories between them.

Publications mainly originate from three source domains—Ecology/Earth/Marine, Physics/Materials/Chemistry, and Mathematics/Systems/Mathematical—and primarily flow into Plant/Ecology/Zoology/Geology/Geophysics, Chemistry/Materials/Physics, and Systems/Computing/Information Science, respectively. These citation paths reveal that Earth sciences provide the primary application context for aerosol deposition and glacier-response research; physics and chemistry underpin analyses of optical properties, radiative transfer, and physicochemical processes; and computational disciplines support retrieval algorithms, data assimilation, and modeling. Overall, the dual-map overlay highlights a natural-science-centered knowledge backbone, with comparatively fewer connections to environmental health or socio-economic domains, pointing to opportunities for future interdisciplinary expansion. Consistent with these journal-level transfer patterns, the subsequent cluster dependencies analysis further delineates how thematic clusters rely on Earth-science foundations, physical/chemical mechanisms, and computational methods.

#### Cluster dependencies

**Figure 9:**
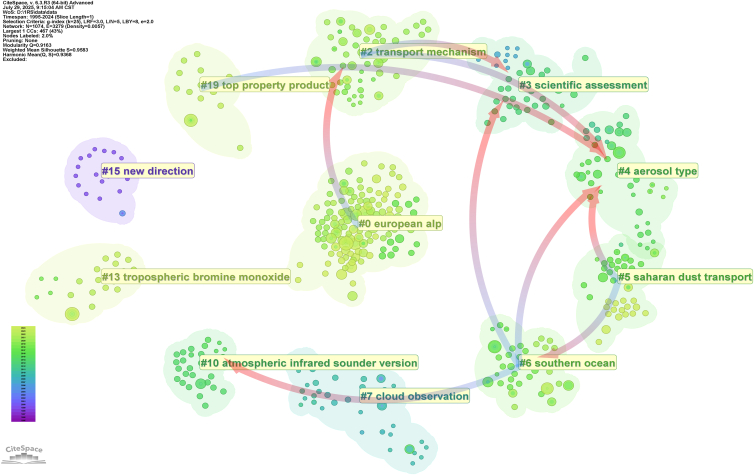
Cluster dependency network of remote sensing studies on aerosol-glacier interactions (1995–2024) This co-citation cluster dependency network illustrates hierarchical relationships among major knowledge clusters. Nodes represent clusters, and directed links indicate dependency pathways. Network quality is quantified using modularity (Q) and weighted mean silhouette value (S). Q, modularity; S, silhouette value. Overall, this figure highlights aerosol type and scientific assessment as the most fundamental clusters structuring knowledge development.

The co-citation cluster dependency network exhibits a well-defined structure with excellent clustering quality (Figure 9), characterized by a modularity *Q* = 0.9163 and weighted mean silhouette *S* = 0.9583, indicating high reliability and thematic consistency of the identified clusters. The dependency analysis reveals a relatively clear hierarchical chain. First, #0 European alp as a frontier cluster depends on the foundational results of #2 transport mechanism, indicating that regional glacier-atmosphere studies must be built upon an understanding of transport mechanisms. Cluster #2 itself connects to #3 scientific assessment and #4 aerosol type, showing that transport mechanism research serves both as a key source for scientific assessment frameworks and as theoretical support for aerosol classification. At the same time, #5 Saharan dust transport points to #4 and #6 Southern Ocean, suggesting that the study of regional dust transport relies on the methodological foundation of aerosol classification and specific environmental research. Cluster #6 further connects to #3, #4, and #10 atmospheric infrared sounder versions, implying that research in the Southern Ocean not only provides empirical validation for scientific assessments and aerosol classification but also promotes the development of infrared detection techniques. Finally, #19 top property product also points to #4, emphasizing that research on remote sensing products methodologically depends on aerosol classification systems.

Overall, #4 aerosol type and #3 scientific assessment emerge as the most central foundational clusters, serving as convergence points of multiple dependency chains. These dependencies reveal a clear developmental trajectory of the field: starting from regional case studies and product development (#0, #5, #19), transitioning through mechanism-oriented research (#2) and specific environmental investigations (#6), and ultimately converging upon the fundamental frameworks of aerosol classification (#4) and scientific assessment (#3). This dependency structure indicates that only when aerosol classification and assessment standards are sufficiently consolidated can regional studies, product development, and transport mechanism research achieve cumulative academic value. These findings, supported by the high Q and S values, confirm that the network structure robustly captures the intellectual evolution of the field, suggesting that future studies should further consolidate these core clusters while expanding methodological and regional frontiers.

The results from Figures 2, 3, 4, 5, 6, 7, 8, and 9 demonstrate that the field’s structural and thematic landscape has undergone profound transformation over the past three decades. The evolution of knowledge in aerosol-glacier interaction research reflects a clear transition from fragmented empirical exploration to an integrated, mechanism-oriented system. The geographical expansion of collaboration has facilitated data accessibility and cross-regional validation, while thematic convergence has driven the coupling of atmospheric and cryospheric processes under a unified physical framework. Meanwhile, methodological innovation—from remote-sensing retrievals to process modeling and machine learning—has deepened quantitative understanding and enabled multi-scale assessment. This co-evolution of spatial cooperation, thematic integration, and technological advancement marks the maturation of the field into a data-intensive and interdisciplinary research paradigm, providing a scientific foundation for future climate and glacier-change assessments.

This study provides a comprehensive bibliometric analysis of remote sensing research on aerosol-glacier interactions from 1995 to 2024, covering 523 publications. The results indicate a continuous growth of scientific output, especially after 2013, accompanied by the formation of a stable research community and enhanced international collaboration. The global cooperation network displays a “North America–Europe–Asia” tripolar pattern, with the United States and European countries maintaining scientific leadership, while China has become an increasingly influential contributor. At the institutional and author levels, collaboration clusters demonstrate both strong expertise concentration and regional fragmentation, reflecting the coexistence of academic maturity and the need for deeper cross-regional integration.

Thematic and structural analyses reveal a clear research evolution. Keyword clustering identifies four dominant research directions—from observation and retrieval methods, to glacier-deposition coupling, snow-ice energy balance, and large-scale AOD-polar linkages—while the keyword time zone visualization highlights the chronological shift of hotspots from atmospheric observation to black carbon, albedo, and regional energy-balance studies. The journal dual-map overlay and cluster dependency analyses further indicate that remote sensing-based aerosol-glacier research is supported by three disciplinary pillars—Earth sciences, physics-chemistry, and computational systems. The interconnection of these clusters illustrates the transition of the field from data acquisition and retrieval toward integrated modeling, process interpretation, and application to glacier-climate feedbacks.

Furthermore, the alignment between bibliometric hotspots and global open-climate data initiatives (e.g., ESA CCI, NASA Earthdata, and NSIDC) demonstrates the co-evolution of scientific priorities and data-sharing standards, underscoring the importance of transparent and standardized datasets for advancing aerosol-cryosphere research and supporting climate assessments. Looking ahead, universities and research centers should focus on enhancing multi-source data fusion, coupling energy-balance and mass-balance modeling, and improving the quantification of light-absorbing aerosol impacts across diverse climatic regions such as the Tibetan Plateau, the Arctic, and the Andes. Building interoperable databases and integrating remote-sensing observations with process-based models will enable more consistent cross-regional comparisons and long-term monitoring of glacier-aerosol interactions. For funding agencies and foundations, long-term interdisciplinary observation programs are essential to connect field monitoring, model development, and policy assessment. Investment should prioritize sustained observation networks, open-data infrastructures, and collaborative research platforms that facilitate both scientific advancement and practical applications. Strengthening these efforts will accelerate the transition of remote sensing from a diagnostic observation tool into a proactive decision-support framework for glacier protection and sustainable cryosphere management—thereby realizing the study’s vision of moving “from observation to protection.”

### Limitations of the study

This study, while providing a comprehensive bibliometric overview of aerosol-glacier interaction research, has certain scope-related limitations. First, the analysis is based on the Web of Science Core Collection, which ensures data consistency but may exclude some relevant work published in other databases or non-English outlets. Second, the accuracy of collaboration and keyword analyses is influenced by metadata quality, which can occasionally lead to an incomplete representation of research networks. Third, as a bibliometric synthesis, this study emphasizes knowledge structure and research trends rather than evaluating the quality of individual studies or conducting direct empirical assessment. Even so, the findings offer valuable perspectives that can complement future efforts combining bibliometric analysis with observational data, modeling approaches, and interdisciplinary studies.

## Resource availability

### Lead contact

Further information and requests for resources and materials should be directed to and will be fulfilled by the lead contact, Feiteng Wang (wangfeiteng@lzb.ac.cn).

### Materials availability

This study did not generate new unique materials.

### Data and code availability

• All data reported in this article are available upon request from the lead contact.
• This article does not report original code.
• Any additional information required to reanalyze the data reported in this article is available from the lead contact upon request.

## Acknowledgments

This work was supported by the China Railway Group Limited Science and Technology Research and Development Plan (Academy of Scientific Research, 2023-Major-01); the Science and Technology Project of TPRI (Grant No. 092317-102); the 10.13039/501100001809National Natural Science Foundation of China (Grant No. 42301166); and the 10.13039/501100004775Natural Science Foundation of Gansu Province (Grant No. 23JRRA658).

## Author contributions

Conceptualization, H.M. and S.L.; methodology, H.M. and X.J.; software, H.M.; validation, H.M. and X.J.; formal analysis, H.M. and X.J.; data curation, S.L., M.X., and F.W.; visualization, H.M. and X.J.; investigation, S.L., M.X., and F.W.; resources, J.M. and F.W.; writing – original draft, H.M.; writing – review and editing, H.M., S.L., X.J., M.X., F.W., and J.M. All authors read and approved the final article.

## Declaration of interests

The authors declare no competing interests.

## STAR★Methods

### Key resources table

**Table:** 

REAGENT or RESOURCE	SOURCE	IDENTIFIER
**Deposited data**		

Bibliographic records on aerosol–glacier remote sensing (1995–2024)	Web of Science Core Collection (SCI-E, SSCI)	Retrieved on June 10, 2025
Cleaned and analyzed bibliometric dataset	This paper	Available upon request from the lead contact
Thesaurus files for data cleaning and standardization	This paper	Provided in supplemental information

**Software and algorithms**		

CiteSpace (v6.3.R3)	Drexel University	https://citespace.podia.com
VOSviewer (v1.6.20)	Leiden University	https://www.vosviewer.com
Topological Data Analysis (TDA)	Custom implementation	Implemented as described in method details
Pajek	University of Ljubljana	http://mrvar.fdv.uni-lj.si/pajek
Scimago Graphica	SCImago	https://www.graphica.app
Microsoft Excel	Microsoft	https://www.microsoft.com/excel
Origin 2021	OriginLab	https://www.originlab.com
Adobe Illustrator	Adobe	https://www.adobe.com/products/illustrator.html

**Other**		

Bibliometric analysis workflow and parameters	This paper	Described in method details
Thesaurus file for field cleaning (keywords, countries, institutions, authors)	This paper	Provided as supplemental information

### Experimental model and study participant details

This bibliometric study does not involve experimental models or human/animal participants.

### Method details

#### Data sources

The data used in this study were obtained from the Web of Science (WoS) Core Collection, including the Science Citation Index Expanded (SCI-E) and the Social Sciences Citation Index (SSCI). Owing to its broad coverage and comprehensive citation indexing, this database is widely applied in bibliometric research, allowing for systematic tracking of the development trajectory and scholarly communication within relevant disciplines. The search query was defined as follows:

TS=((remote sensing OR remote sensing technolog∗ OR remote sensing monitoring OR satellite remote sensing OR satellite monitoring OR satellite imagery OR geographic information system∗ OR GIS OR synthetic aperture radar OR SAR OR interferometric synthetic aperture radar OR InSAR OR MODIS OR moderate resolution imaging spectroradiometer OR Sentinel∗ OR LiDAR OR laser radar OR Landsat∗ OR ASTER OR VIIRS OR NSIDC OR AMSR-E OR satellite product∗) AND (aerosol∗ OR atmospheric particulate∗ OR particulate matter OR black carbon OR BC OR brown carbon OR PM2.5 OR PM10 OR dust OR dust storm OR atmospheric dust OR windblown dust OR fugitive dust OR deposition OR aerosol optical depth OR AOD OR aerosol optical thickness OR AOT) AND (glacier∗ OR glacier ablation OR glacier melt∗ OR glacier retreat OR glacier mass balance OR glacier change∗ OR ice cap∗)).

The data were retrieved on June 10, 2025. The document types were limited to articles, reviews, and proceedings, as these three categories accounted for more than 85% of the total retrieval results.18 A total of 528 records were initially retrieved. To ensure data accuracy and consistency, the dataset was cleaned by removing two Early Access records with publication year (PY) = 2025 and three duplicates, resulting in a total of 523 valid publications. No In Press items were included in the final dataset. To ensure dataset relevance, a random validation was conducted by manually checking 10% of the retrieved records to confirm thematic consistency with the topic “remote sensing, atmospheric aerosols, and glacier change.” All sampled records were verified to fall within the research scope, indicating no false-positive results in the final dataset.

#### Methods

Multiple bibliometric and visualization tools were employed to analyze the knowledge structure and research frontiers of the field. Data deduplication and field cleaning were carried out using CiteSpace (version 6.3.R3) and TDA. Duplicate records were identified by cross-checking titles, DOIs, and publication years through automated detection and manual verification. A thesaurus automatically generated by TDA was applied for field cleaning and manually refined to ensure accuracy. The thesaurus was used to standardize keywords, countries, institutions, and author names, ensuring consistency in collaboration and co-occurrence analyses. All thesaurus information has been provided in the Supplemental Information for reproducibility. Data processing and figure production primarily relied on Microsoft Excel, Origin 2021, and Adobe Illustrator to ensure standardized presentation and high-quality visualization. For specific analyses, VOSviewer (version 1.6.20) and Scimago Graphica were used to visualize national and regional cooperation; institutional and author collaboration networks were constructed with VOSviewer; highly cited articles were analyzed using TDA; keyword clustering was conducted by combining VOSviewer and Pajek; while burst detection, keyword timezone, dual-map overlay of journals, and cluster dependency analyses were performed with CiteSpace. The bibliometric workflow followed established methodological frameworks in science mapping,19,20 ensuring methodological transparency and reproducibility.

#### Research framework

The research framework of this study consists of four components: (1) analyzing publication output to reveal the temporal dynamics of the field; (2) constructing cooperation networks of countries, institutions, and authors to identify major contributors and collaboration structures; (3) examining journal distribution, highly cited literature, keyword clusters, and burst terms to identify research hotspots and their temporal evolution; and (4) combining journal dual-map overlays with cluster dependency analysis to explore the knowledge bases, disciplinary migration paths, and thematic dependencies within the field. Through this framework, the study provides a comprehensive understanding of the overall landscape and developmental trajectory of remote sensing applications in aerosol–glacier interaction research.

### Quantification and statistical analysis

Quantitative analyses in this study were conducted using bibliometric and network-based indicators rather than traditional hypothesis-testing statistics. Publication and citation trends were quantified using annual counts, cumulative frequencies, and a quadratic fitting of yearly publication output to derive the coefficient of determination (R^2^) as a measure of goodness of fit. For countries, institutions, and authors, total publications, total citations, average citations per paper, average publication year, and H-index were calculated following standard bibliometric definitions and summarized in the corresponding tables.

Collaboration and knowledge networks were analyzed using standard network metrics, including node degree, total link strength, betweenness centrality, and network density, to characterize the structure and intensity of cooperation at national, institutional, and author levels. Keyword co-occurrence and co-citation analyses were performed to identify major research themes and intellectual structures, and cluster quality was evaluated using modularity (Q) and silhouette values, with higher values indicating clearer community structure and greater cluster homogeneity. Citation burst detection was applied to keywords to identify emerging research topics and periods of increased attention, and timezone visualizations were used to track the temporal evolution of hotspot themes.

All quantitative analyses and visualizations were implemented using CiteSpace (v6.3.R3), VOSviewer (v1.6.20), Pajek, and Scimago Graphica, as described in the method details section. Parameter settings for thresholding, time-slicing, and clustering followed recommended defaults for bibliometric analysis unless otherwise specified in the figure legends.

## Supplemental information

Document S1. Figures S1 and S2 and Tables S1–S5 and S7

Table S6. Thesaurus used to standardize author names for consistent author-level collaboration and citation analysis in aerosol-glacier remote sensing bibliometric studies
