# Supplementary material for: From observation to protection a bibliometric analysis of aerosol-glacier interactions
Source: iScience. 2026 Jan 30;29(3):114672. doi: 10.1016/j.isci.2026.114672 (PMC12925231; doi:10.1016/j.isci.2026.114672)
Supplement: Document S1. Figures S1 and S2 and Tables S1–S5 and S7 [file mmc1.pdf]

iScience, Volume *29*

Supplemental information

From observation to protection a bibliometric analysis of aerosol-glacier interactions

Hongfei Meng, Feiteng Wang, Shuangshuang Liu, Xiang Jin, Mengwei Xu, and Jianxin Mu

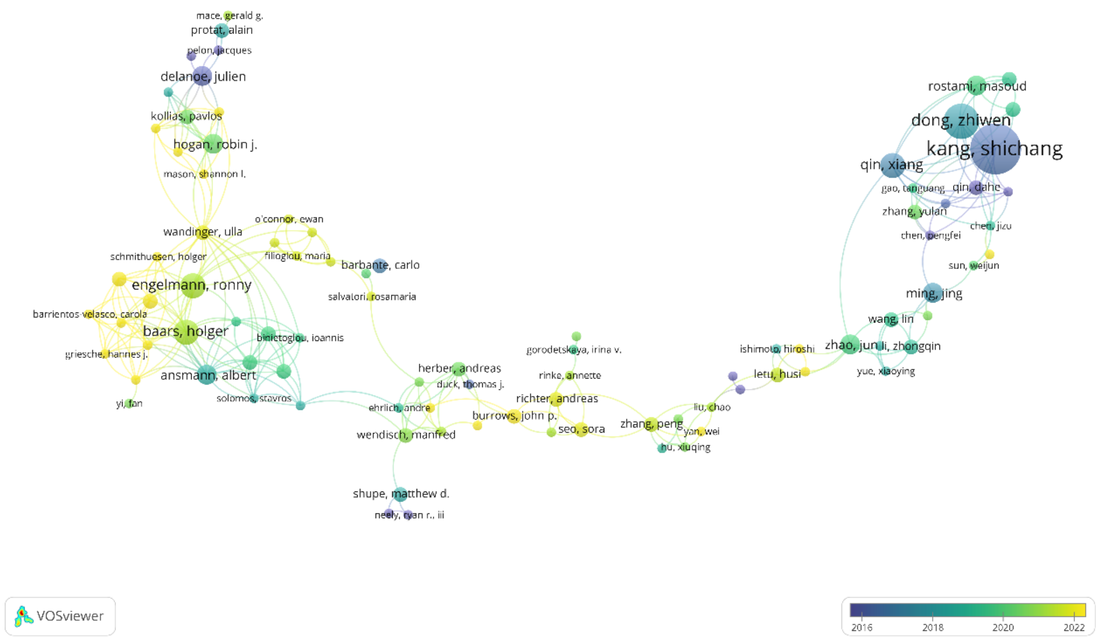

Figure S1: Visualization of global author collaboration patterns in remote sensing studies of aerosol–glacier interactions from 1995 to 2024.

The network visualization generated using VOSviewer illustrates collaboration clusters among authors. Node size reflects publication output, link strength indicates co-authorship frequency, and color represents the average year of publication.

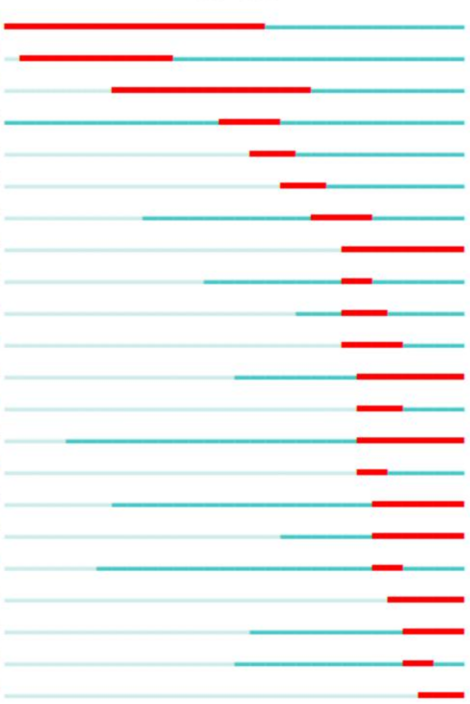

Top 22 Keywords with the Strongest Citation Bursts

Keywords	Year	Strength	Begin	End	1995 - 2024
general circulation model	1995	2.49	**1995**	2011	
atmosphere	1996	2.67	**1996**	2005	
cirrus clouds	2002	2.59	**2002**	2014	
climate	1995	3.03	**2009**	2012	
particles	2011	3.3	**2011**	2013	
retrieval	2013	3.39	**2013**	2015	
transport	2004	2.66	**2015**	2018	
mass balance	2017	4.12	**2017**	2024	
lidar	2008	2.66	**2017**	2018	
snow	2014	2.56	**2017**	2019	
radiative property	2017	2.5	**2017**	2020	
black carbon	2010	5	**2018**	2024	
optical depth	2018	2.75	**2018**	2020	
greenland ice sheet	1999	2.53	**2018**	2024	
emissions	2018	2.45	**2018**	2019	
glacier	2002	2.9	**2019**	2024	
tibetan plateau	2013	2.76	**2019**	2024	
deposition	2001	2.5	**2019**	2020	
snow albedo	2020	2.84	**2020**	2024	
model	2011	2.79	**2021**	2024	
modis	2010	2.41	**2021**	2022	
calipso	2022	3.35	**2022**	2024	

Figure S2: Temporal patterns of research hotspots identified through citation burst analysis of keywords in aerosol–glacier remote sensing studies from 1995 to 2024.

The figure presents citation burst detection results generated using CiteSpace for the top 22 keywords with the strongest citation bursts. Red bars indicate the periods of significant citation bursts, while the blue line represents the overall time span.

Table S1: Summary statistics of the top 30 most highly cited publications in remote sensing studies of aerosols and glaciers from 1995 to 2024.

Title	Publication Year	Total Citations
Discriminating clear sky from clouds with modis	1998	981
Prelaunch characteristics of the moderate resolution imaging spectroradiometer (modis) on eos-am1	1998	712
Improving our fundamental understanding of the role of aerosol-cloud interactions in the climate system	2016	527
Early on-orbit performance of the visible infrared imaging radiometer suite onboard the suomi national polar-orbiting partnership (s-npp) satellite	2014	441
The earthcare satellite the next step forward in global measurements of clouds, aerosols, precipitation, and radiation	2015	427
The esa climate change initiative satellite data records for essential climate variables	2013	374
Precipitation in the hindu-kush karakoram himalaya: observations and future scenarios	2013	366
The whole atmosphere community climate model version 6 (waccm6)	2019	365
Arctic smoke -: record high air pollution levels in the european arctic due to agricultural fires in eastern europe in spring 2006	2007	322
The esa medium resolution imaging spectrometer meris -: a review of the instrument and its mission	1999	313
Saharan dust storms and indirect aerosol effects on clouds: crystal-face results	2003	282
Illuminating the capabilities of the suomi national polar-orbiting partnership (npp) visible infrared imaging radiometer suite (viirs) day/night band	2013	280
Application of a 3d laser scanner in the assessment of erosion and deposition volumes and channel change in a proglacial river	2007	242
The fog remote sensing and modeling field project	2009	231
Mountain lakes: eyes on global environmental change	2019	220
The tibetan plateau cryosphere: observations and model simulations for current status and recent changes	2019	213

Slope instability in relation to glacial debuttressing in alpine areas (upper durance catchment, southeastern france):: evidence from field data and ¹⁰be cosmic ray exposure ages	2008	198
New directions in earth observing: scientific applications of multiangle remote sensing	1999	196
First-light imagery from suomi npp viirs	2013	189
State of the antarctic and southern ocean climate system	2009	187
Overview of the mosaic expedition-atmosphere introduction	2022	183
Sequence of infilling events in gale crater, mars: results from morphology, stratigraphy, and mineralogy	2013	160
Remote sensing of liquid water and ice cloud optical thickness and effective radius in the arctic: application of airborne multispectral mas data	2004	160
Atmospheric brown clouds reach the tibetan plateau by crossing the himalayas	2015	159
Mineralogy of saharan dust transported over northwestern tropical atlantic ocean in relation to source regions	2002	156
Miniature thermal emission spectrometer for the mars exploration rovers	2003	154
Direct-estimation algorithm for mapping daily land-surface broadband albedo from modis data	2014	151
Observations of clouds, aerosols, precipitation, and surface radiation over the southern ocean: an overview of capricorn, marcus, micre, and socrates	2021	147
Small-scale temperature variations in the vicinity of nlc:: experimental and model results -: art. No. 4392	2002	147
Response of phytoplankton dynamics to 19-year (1991-2009) climate trends in potter cove (antarctica)	2012	143

Table S2: Comparative statistics of publication output, citation impact, and average publication year for the top 22 countries and regions in aerosol–glacier remote sensing research from 1995 to 2024.

Countries	Documents	Total Citations	Average Citations per Paper	Average Publication Year
United States	227	16398	72.23788546	2014.9824
China	102	7836	76.82352941	2018.8529
Germany	95	8778	92.4	2017.5158
United Kingdom	70	9117	130.2428571	2015.7429
France	68	3667	53.92647059	2016.9853
Canada	46	2026	44.04347826	2017.8478

Switzerland	44	6385	145.1136364	2017.5909
Italy	43	1738	40.41860465	2017.4884
India	33	4997	151.4242424	2017.6061
Norway	31	6008	193.8064516	2016.9677
Japan	26	6182	237.7692308	2016.3846
Netherlands	25	1519	60.76	2016.6
Australia	22	1037	47.13636364	2017.3636
Finland	18	1277	70.94444444	2018
Sweden	18	887	49.27777778	2016.2778
Austria	17	5578	328.1176471	2016.4706
Russia	15	397	26.46666667	2016.9333
Belgium	14	1002	71.57142857	2019.7857
Poland	13	216	16.61538462	2020.3846
Spain	13	758	58.30769231	2016.6154
Iceland	12	237	19.75	2018.0833
Denmark	11	733	66.63636364	2017.5455

Table S3: Comparative statistics of publication output, citation impact, and average publication year for the top institutions with at least 10 publications in aerosol–glacier remote sensing research from 1995 to 2024.

Institutions	Documents	Total Citations	Average Citations per Paper	Average Publication Year
NASA	56	4104	73.29	2012.29
Chinese Acad Sci	48	1843	38.40	2018.29
Univ Colorado	30	1220	40.67	2015.10
Caltech	25	1690	67.60	2013.36
Noaa	19	1587	83.53	2017.11
Colorado State Univ	17	1991	117.12	2015.59
Univ Chinese Acad Sci	15	327	21.80	2020.47
Univ Bremen	14	575	41.07	2019.71
Cnr	14	469	33.50	2017.71
Us Geol Survey	13	758	58.31	2015.92
Lanzhou Univ	13	319	24.54	2020.23
Univ Wisconsin	12	2092	174.33	2011.33
Univ Leeds	12	1280	106.67	2016.33
Univ Washington	12	1023	85.25	2017.58
Texas A&M Univ	12	490	40.83	2014.83
Wuhan Univ	12	245	20.42	2019.75
Univ Maryland	11	811	73.73	2013.82
Natl Ctr Atmospher Res	11	760	69.09	2016.82
Univ Reading	11	697	63.36	2016.55

Univ Calif Los Angeles	11	593	53.91	2008.91
Finnish Meteorol Inst	10	665	66.50	2017.10
Sci Syst & Applicat Inc	10	644	64.40	2013.30
Univ Maryland Baltimore Cty	10	548	54.80	2012.50
Univ Arizona	10	376	37.60	2013.50
Univ Iceland	10	229	22.90	2017.00
Univ Grenoble Alpes	10	118	11.80	2021.20

Table S4: Thesaurus used to standardize country and region names for consistent bibliometric analysis in aerosol–glacier remote sensing research.

**label**	**replace by**
Taiwan	China
peoples r china	China
the people's republic of china	China
PEOPLES R CHINA	China
England	United Kingdom
Scotland	United Kingdom
Wales	United Kingdom
Northern Ireland	United Kingdom
UK	United Kingdom
West Germany	Germany
The United States of America	United States
United States	United States
USA	United States

Table S5: Thesaurus used to standardize institutional names for consistent affiliation analysis in aerosol–glacier remote sensing bibliometric studies.

**label**	**replace by**
Belgian Inst Space Aeron BIRA IASB	Belgian Inst Space Aeron BIRA IASB
Belgian Inst Space Aeron	Belgian Inst Space Aeron BIRA IASB
Bur Meteorol Res Ctr	Bur Meteorol
Bur Meteorol	Bur Meteorol
Indian Inst Technol Kharagpur	Indian Inst Technol
Indian Inst Technol	Indian Inst Technol
Japan Meteorol Agcy	Japan Meteorol Agcy
Japan Meteorol Agency	Japan Meteorol Agcy
Natl Agcy Meteorol	Nigerian Meteorol Agcy
Nigerian Meteorol Agcy	Nigerian Meteorol Agcy
NOAA NESDIS STAR Univ Wisconsin	NOAA NESDIS STAR

NOAA NESDIS STAR	NOAA NESDIS STAR
Univ British Columbia	Univ British Columbia
Univ No British Columbia	Univ British Columbia
Univ Sci & Technol China USTC	Univ Sci & Technol China
Univ Sci & Technol China	Univ Sci & Technol China

Table S7: Thesaurus used to standardize keywords for consistent keyword co-occurrence analysis in aerosol–glacier remote sensing bibliometric studies.

**label**	**replace by**
aerosol	Aerosols
Aerosols	Aerosols
Aerosols/ particulates	Aerosols/ particulates
Aerosols/particulates	Aerosols/ particulates
Ash dispersal	Ash dispersal
Ash dispersion	Ash dispersal
cloud	Clouds
Clouds	Clouds
Database	Databases
Databases	Databases
De Geer moraine	De Geer moraines
De Geer moraines	De Geer moraines
Debris flow	Debris flow
Debris flows	Debris flow
disaster chain	Disaster chains
Disaster chains	Disaster chains
Driving factor	Driving factor
driving factors	Driving factor
drumlin	drumlin
drumlinization	drumlin
Drumlins	drumlin
Dust aerosol	Dust aerosol
Dust aerosols	Dust aerosol
dust storm	Dust storms
Dust storms	Dust storms
Field campaign	field campaigns
field campaigns	field campaigns
flood	Floods
Flooding	Floods
Floods	Floods
General Circulation Model	General Circulation Model
general circulation models	General Circulation Model
Geomorphic change	Geomorphic changes

Geomorphic changes	Geomorphic changes
glacial lake	glacial lake
glacial lakes	glacial lake
glacier	glacier
glaciers	glacier
Glacier surface albedo	Glacier surface albedo
Glacier-surface albedo	Glacier surface albedo
Glacier surge	Glacier surge
glacier surging	Glacier surge
gravity wave	gravity waves
gravity waves	gravity waves
Ground Penetrating Radar	Ground Penetrating Radar
ground-penetrating radar	Ground Penetrating Radar
Heating rate	Heating rates
Heating rates	Heating rates
Himalayan glacier	Himalayan glacier
Himalayan glaciers	Himalayan glacier
Himalaya	Himalayas
Himalayas	Himalayas
hyperspectral imagers	hyperspectral imagers
hyperspectral images	hyperspectral imagers
ice	ice
Ices	ice
ice cloud	ice cloud
ice clouds	ice cloud
Ice core	Ice core
Ice cores	Ice core
In situ measurements	In-situ measurements
In-situ measurements	In-situ measurements
Jokulhlaup	Jokulhlaup
Jokulhlaups	Jokulhlaup
lidar	lidar
Lidars	lidar
lidar observation	lidar observation
Lidar observations	lidar observation
Light absorbing impurities	Light-absorbing impurities
Light-absorbing impurities	Light-absorbing impurities
light-absorbing impurity	Light-absorbing impurities
Mars climate	Mars, Climate
Mars, Climate	Mars, Climate
Mixed-phase cloud	mixed-phase clouds
mixed-phase clouds	mixed-phase clouds
modeling	modeling

modelling	modeling
mountain glacier	mountain glaciers
mountain glaciers	mountain glaciers
natural hazard	natural hazard
Natural hazards	natural hazard
polar	Polarization
Polarization	Polarization
Proglacial area	proglacial areas
proglacial areas	proglacial areas
reflectance	Reflectivity
Reflectivity	Reflectivity
Repeat TLS surveys	Repeat TLS surveys
repeated TLS surveys	Repeat TLS surveys
Rock glacier	Rock glacier
Rock glaciers	Rock glacier
satellite	satellite
satellites	satellite
satellite surface	satellite surface
surfaces, satellites	satellite surface
sediment	sediment
Sedimentation	sediment
Sediment budget	Sediment budget
Sediment budgeting	Sediment budget
sediment budgets	Sediment budget
Slope failure	Slope failure
Slope-failure	Slope failure
Rough surfaces	Surface roughness
Surface roughness	Surface roughness
spatial and temporal variation	temporal and spatial variations
temporal and spatial variations	temporal and spatial variations
Trace element	trace elements
trace elements	trace elements
Volcanic eruption	Volcanic eruption
volcanic eruptions	Volcanic eruption
wildfire	wildfire
wildfires	wildfire
